# Antimicrobial Resistance Profiles of Multidrug-Resistant Pathogens in a Military Hospital: A Three-Year Study

**DOI:** 10.7759/cureus.91297

**Published:** 2025-08-30

**Authors:** Dimitra Stamkou, Eleftherios Panteris, Panagiotis Siasios, Dimitra Antoniou, Zoumpoulia Vaitsidou

**Affiliations:** 1 Microbiology Laboratory, 424 General Military Hospital (424 GMHT), Thessaloniki, GRC; 2 Biomedical Sciences, Metropolitan College, Campus of Thessaloniki, Thessaloniki, GRC; 3 Neonatology/Neonatal Intensive Care Unit (NICU), University General Hospital of Heraklion, University of Crete, Heraklion, GRC; 4 Financial and Management Engineering, University of the Aegean, Chios, GRC

**Keywords:** antimicrobial resistance, carbapenem-resistant, hospital-acquired infections, klebsiella pneumoniae (kp), multidrug-resistant microorganisms, pseudomonas aeruginosa, public health threat, resistance mechanisms, super bugs, therapeutic options

## Abstract

The global rise of multidrug-resistant (MDR) microorganisms poses a significant threat to public health by limiting effective treatment options. This study aimed to assess the incidence, resistance profiles, and distribution of MDR organisms within the 424 General Military Training Hospital in Thessaloniki, Greece, over a three-year period (2021-2023). A total of 7,856 microbial isolates were collected, of which 131 were confirmed as MDR and analyzed further. Isolates were obtained from a variety of clinical specimens, including blood, urine, bronchial lavage, sputum, catheters, and wound secretions, originating from several hospital departments such as the ICU, emergency department, and general surgery. Microbial identification and antimicrobial susceptibility testing were conducted using the Vitek 2-Compact system (BioMérieux, Marcy-l'Étoile, France), following Clinical and Laboratory Standards Institute (CLSI) guidelines.

*Klebsiella pneumoniae* was the most frequently isolated MDR pathogen, followed by *Acinetobacter baumannii* and *Pseudomonas aeruginosa*. Notably, *Klebsiella pneumoniae* demonstrated increasing resistance over time, especially against carbapenems and third-generation cephalosporins. *Acinetobacter baumannii* showed persistently high resistance, while *Pseudomonas aeruginosa* maintained a more moderate profile. Urine and blood were the most common sources of MDR isolates, with *Klebsiella pneumoniae* predominating. *Acinetobacter baumannii* was more often found in respiratory samples, whereas *Pseudomonas aeruginosa* appeared mostly in wound cultures. The ICU and emergency department were identified as key locations for MDR pathogen isolation. Although monthly variations were observed, no consistent seasonal trends were noted.

These findings emphasize the urgent need for targeted antimicrobial stewardship, stricter infection control practices, and continuous surveillance to prevent further escalation of MDR infections in healthcare settings.

## Introduction

Multidrug-resistant (MDR) microbes are microorganisms that have developed resistance to multiple antibiotics, rendering these drugs ineffective in treating infections caused by these pathogens. This resistance significantly restricts the available therapeutic options for controlling or eradicating these microorganisms, often necessitating the use of last-resort agents such as colistin or tigecycline. [1,2]. On the European Antibiotic Awareness Day, 18 November, the European Center for Disease Prevention and Control (ECDC), in collaboration with the World Health Organization (WHO), highlighted that every year 35,000 people die from infection due to antimicrobial-resistant bacteria [3]. Antimicrobial resistance has increased globally, affecting both developed and developing countries [4, 5]. 

The excessive and inappropriate use of antimicrobial agents has contributed to the widespread occurrence of mainly hospital-acquired infections caused by MDR microorganisms [6, 7, 8]. The overuse of antibiotics not only disrupts the body’s normal microflora but also accelerates the development of drug-resistant infections, which can ultimately become untreatable [9].

This phenomenon poses a significant challenge in Greece, which has some of the highest resistance rates in Europe. Compounding this issue, Greece also ranks first in antimicrobial consumption across Europe, further fueling the problem [10]. According to the ECDC, approximately 2,000 people die every year in Greece from infections caused by antibiotic-resistant microorganisms. The spread of MDR strains poses a daily challenge, as these strains are highly infectious, spread rapidly, and cause severe infections in critically ill patients, resulting in high mortality rates [11]. Although MDR pathogens are often associated with nosocomial transmission, this study did not directly assess their spread or transmissibility.

MDR bacteria exhibit various mechanisms that allow them to evade the action of antibiotics. These mechanisms include efflux pumps, alterations in drug-binding sites, changes in membrane permeability, and the production of degradation enzymes. Additionally, bacteria can modify antibiotics through enzymatic processes such as phosphorylation, acetylation, adenylation, and/or hydrolysis, leading to drug inactivation. These combined strategies significantly hinder the efficacy of many antimicrobial agents used in clinical practice [12, 13]. Bacterial resistance can be generated by one of the above mechanisms, either by the accumulation of multiple genes, each encoding resistance to a single antibiotic, within a single cell, which is usually facilitated by resistance plasmids, or by the overexpression of genes encoding multidrug efflux pumps, which actively expel antibiotics from the bacterial cell, resulting in decreased susceptibility to a broad range of antimicrobial agents [14].

Despite extensive research on the resistance mechanisms, familiarity with the optimal treatment for infections caused by MDR organisms remains limited. Neither monotherapy nor combination therapy has been consistently proven effective, highlighting the need for individualized treatment plans. Considering the pharmacokinetics and pharmacodynamics of the antibiotics on a case-by-case basis is essential for ensuring more effective therapeutic outcomes [15, 16].

This lack of effective treatment options is particularly concerning given the prevalence of specific infections caused by these resistant microorganisms. Common infections caused by MDR microorganisms include urinary tract infections, pneumonia, bacteremia, and wound infections. Particularly, widespread MDR microbes include the Gram-negative bacteria *Pseudomonas aeruginosa*, *Acinetobacter baumannii*, *Klebsiella pneumoniae*, *Escherichia coli*, and *Enterobacter* spp. [17, 18]. These pathogens significantly weaken the most potent class of antibiotics, carbapenems, by producing carbapenemases, enzymes that make them resistant to this class of antibiotics [12,19].

Given the increasing resistance to carbapenems and other antibiotics, studying and understanding the mechanisms of resistance is essential. Identifying the most effective therapeutic approaches, both synergistic and non-synergistic, will help optimize the use of current antimicrobial agents. This, in turn, can reduce hospital stays, lower hospitalization rates, and, ultimately, reduce mortality [20].

Antibiotics included in resistance testing spanned multiple classes, such as beta-lactams (including penicillins, cephalosporins, carbapenems, and monobactams), tetracyclines, quinolones/fluoroquinolones, aminoglycosides, and other antibiotics, such as amphenicol.

Thus, the present study aims to determine the incidence of MDR microorganisms at the 424 General Military Training Hospital, Thessaloniki, Greece, during the period between 2021 and 2023. The study will focus on identifying the biological clinical samples and hospital departments and units where patients are most frequently infected with these microbes, as well as the antibiotics to which these microorganisms exhibit susceptibility and resistance.

## Materials and methods

Study design and sample size

The study analyzed 7,856 clinical isolates obtained from hospitalized patients between 2021 and 2023. These isolates originated from multiple patients; however, the exact number of unique individuals could not be determined, as some patients contributed more than one specimen from different anatomical sites or at different time points. Of the total isolates, 131 were identified as MDR and were further analyzed. The bacterial isolates included in this study were consistent with the WHO priority pathogen list, including *Klebsiella pneumoniae*, *Pseudomonas aeruginosa*, and *Acinetobacter baumannii*. No specific exclusion criteria were applied other than duplicate isolates from the same patient. MDR was defined according to the international consensus by Magiorakos et al. 2012 [21] as acquired non-susceptibility to at least one agent in three or more antimicrobial categories. The types of samples tested included blood, bronchial lavage, urine, trauma-related wound swabs, throat swabs, sputum, percutaneous transluminal drainage (PTD) tips, urinary catheters, surgical drain fluid, and other less frequently encountered specimen types such as synovial fluid and cerebrospinal fluid. Specimens were obtained from multiple hospital departments, including the ICU, surgery, orthopedics, and pathology departments (Appendix A) within the 424 General Military Training Hospital, where only military, police, firefighters, and their first-degree relatives are treated, and the samples were subsequently processed in the microbiological laboratory between 2021 and 2023.

Study measures

The primary step was the clinical study of the cultures to identify microbial growth, followed by identification and antibiogram testing. The process was conducted using the Vitek 2-Compact system (BioMérieux, Marcy-l'Étoile, France), an automated system that provides rapid and accurate microbial identification, along with built-in quality control tests. Antibiotic resistance was determined according to Clinical and Laboratory Standards Institute (CLSI) guidelines [22].

For resistance testing, the Vitek 2-Compact system in accordance with the manufacturer’s instructions was used to assess the susceptibility of microorganisms including agents such as beta-lactams (including penicillins and specifically ampicillin, amoxicillin/clavulanic acid, piperacillin/tazobactam), cephalosporins (including cefalotin, cefuroxime, cefuroxime axetil, cefoxitin, cefpodoxime, cefotaxime, ceftazidime, ceftriaxone, cefepime), carbapenems (including imipenem, meropenem), monobactams (including aztreonam), aminoglycosides (including gentamicin, tobramycin, amikacin), quinolones/fluoroquinolones (including ciprofloxacin, norfloxacin, levofloxacin), tetracyclines (including tetracycline, tigecycline), combination antibiotics (including trimethoprim/sulfamethoxazole) and other antibiotics (including nitrofurantoin, fosfomycin, colistin).

If microbial resistance was detected emerging during the initial antibiotic susceptibility, further tests were investigated and were conducted using an extended panel of antibiotics, including extended-spectrum beta-lactamase (ESBL), which is not an antibiotic but a type of beta-lactamase enzyme produced by bacteria, which confers resistance to a wide range of beta-lactams (penicillins, cephalosporins, and aztreonam). The presence of ESBL implies that these specific antibiotics will not be effective against the microorganism that produces the enzyme: beta-lactams (including penicillins and specifically ampicillin and temocillin), cephalosporins (including cefuroxime, cefuroxime axetil, cefditoren, cefixime, ceftriaxone, ceftazidime/avibactam, and ceftolozane/tazobactam), carbapenems (including imipenem), monobactams (including aztreonam), tetracyclines (including minocycline and tetracycline), quinolones/fluoroquinolones (including levofloxacin and moxifloxacin), aminoglycosides (including tobramycin), and other antibiotics (including chloramphenicol), all processed through the Vitek 2-Compact system.

Antimicrobial susceptibility testing was performed using the Vitek 2-Compact system in accordance with the manufacturer's instructions. In cases where intermediate susceptibility levels (minimum inhibitory concentration (MIC) 3-4 μg/ml) to colistin were observed, particularly in *Enterobacteriaceae*, *Pseudomonas*, and *Acinetobacter *species, the MIC test strip for colistin (COL) method was employed for further evaluation.

For *Klebsiella pneumoniae* strains that tested resistant to beta-lactams on the Vitek 2-Compact system, phenotypic detection of carbapenemases (KPC), metallo-beta-lactamases (MBL), or the co-existence of KPC and MBL was performed using the double-disc synergy test (DDST). Strains that produced these enzymes exhibited reduced susceptibility to carbapenems (MIC >1 μg/ml) and were determined to be resistant to third-generation cephalosporins.

In instances where microorganisms displayed intermediate susceptibility MIC values (3-4 μg/ml) to tigecycline, susceptibility was also evaluated using the E-test method (Diachel, Athens, Greece).

Ethics approval and consent to participate

This research was approved by the relevant institutional and military authorities. Specifically, ethical approval was granted by the Directorate of Health Services, Hellenic Army General Staff, under Protocol No. F.040/28/1416697 - Serial No. 2621, dated 3^rd^ July 2024. The study was conducted at the Microbiology Laboratory of the 424 General Military Hospital of Northern Greece (424 GSNE), in accordance with the ethical principles of the Declaration of Helsinki and the applicable guidelines governing research within military medical facilities.

The Directorate confirmed that no ethical or security objections existed regarding the study, and formal permission for the dissemination of results was granted. All procedures were carried out in compliance with institutional protocols and relevant national legislation.

Data analysis

Data processing was initially done in Microsoft Excel 2019 (Microsoft Corp., Redmond, WA, USA); data manipulations and some visualization procedures were conducted using Python version 3.10 (Python Software Foundation (PSF), Wilmington, DE, USA). The pandas library (version 1.5.3; developed by Wes McKinney and maintained by the open-source community under NumFOCUS) was employed for data manipulation and tabular organization. Numerical computations, such as aggregation and percentage derivation, were performed with NumPy (version 1.24.2; NumPy Steering Council and fiscally supported by U.S.-based nonprofit NumFOCUS). Visualizations were generated using matplotlib (version 3.7.1; originally created by John D. Hunter and now maintained by the Matplotlib Development Team) and IBM SPSS Statistics for Windows, version 26 (IBM Corp., Armonk, NY, USA), which also handled tabulations.

## Results

In 2021, sample numbers ranged between 156 and 246 per month. MDR isolates were detected every month, with high numbers in January (14 isolates) and none in June. *Klebsiella pneumoniae* was observed to be 35.71% of MDR isolates in January. *Pseudomonas aeruginosa* and *Acinetobacter baumannii* were also present, generally accounting for less than 2% of the total samples.

In 2022, monthly samples ranged from 181 to 215 in number, and MDR organisms were identified each month (one to six isolates). *Klebsiella pneumoniae* often represents more than half of the total MDR isolates, such as in January and February (66.67%). *Pseudomonas aeruginosa*, *Acinetobacter baumannii*, and *Providencia stuartii *were also isolated is also represented.

**Figure 1:**
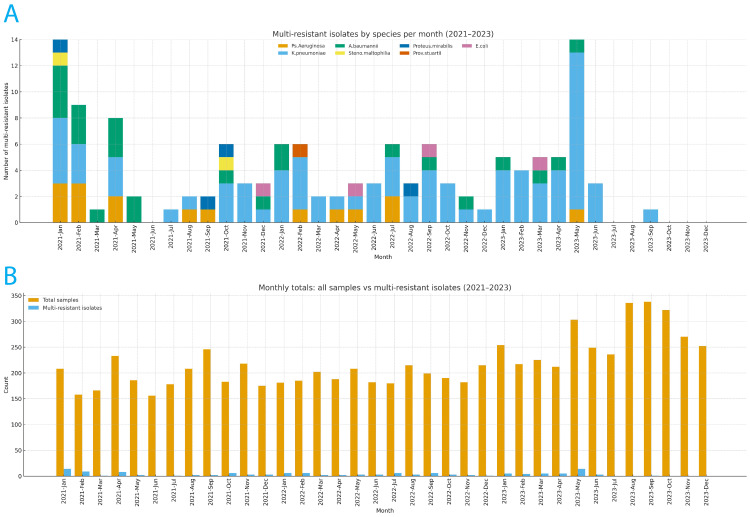
A. Monthly composition of multi-resistant (MR) isolates by species, January 2021–December 2023. Stacked bars show the monthly number of MR isolates, partitioned by species: Pseudomonas aeruginosa, Klebsiella pneumoniae, Acinetobacter baumannii, Stenotrophomonas maltophilia, Proteus mirabilis, Providencia stuartii, and Escherichia coli. The height of each stack equals the MR total for that month. Months without MR detections have a bar of height 0. Counts are plotted (percentages reported in Table 1 are not shown here). B. Monthly totals of samples processed and MR isolates, January 2021–December 2023. Grouped bars compare, for each month, the total number of samples processed and the number of MR isolates detected. Counts are plotted to allow visual comparison of workload and MR burden over time. Ps aeruginosa: Pseudomonas aeruginosa; K. pneumoniae: Klebsiella pneumoniae; A. baumannii: Acinetobacter baumannii; Steno. maltophilia​​​​​​:Stenotrophomonas maltophilia; Prov. stuartii: Providencia stuartii; E. coli: Escherichia coli

**Figure 2:**
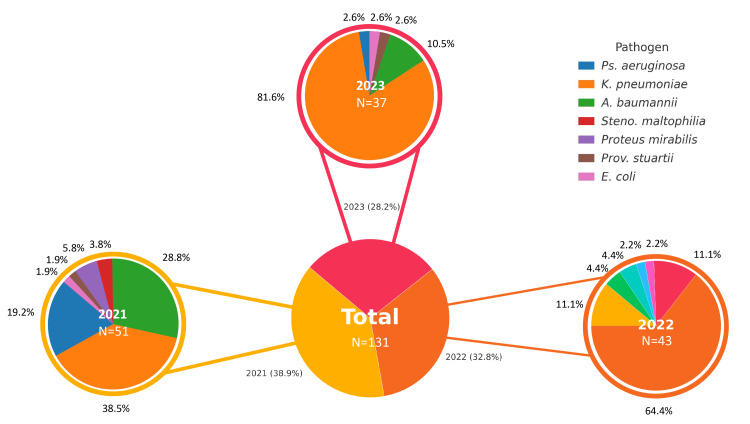
Annual distribution of multidrug-resistant (MDR) microorganisms (2021–2023), with % indicated for each year. MDR pathogens are color-coded according to bacterial species *Ps aeruginosa*: *Pseudomonas aeruginosa*; *K. pneumoniae*: *Klebsiella pneumoniae*; *A. baumannii*: *Acinetobacter baumannii*; *Steno. maltophilia​​​​​​*:*Stenotrophomonas maltophilia*; *Prov. stuartii*: *Providencia stuartii*; *E. coli*: *Escherichia coli*

In 2023, the sample size reached 336 in some months. During the first half of the year (January to June), MDR isolates were observed (typically four to five per month), with a marked peak in May (14 isolates), of which nearly 86% were *Klebsiella pneumoniae*. In this early period, *Klebsiella pneumoniae* consistently accounted for the majority of MDR detections, ranging from 60% to 80% of monthly totals. From July through December, no MDR isolates were reported. Relevant data are presented in Appendix A and Figures 1, 2.

Distribution of MDR pathogens by specimen type

In 2021,* Klebsiella pneumoniae* was primarily isolated from blood (70.0%) and urine (75.0%) samples. *Acinetobacter baumannii *was most frequently detected in PTD extremity specimens (57.1%) and bronchial lavage (50.0%), while *Pseudomonas aeruginosa *was identified predominantly in sputum (100.0%).

In 2022, *Klebsiella pneumoniae* was the most common isolate in blood (83.3%) and urine (66.7%) and was the sole resistant organism detected in pus and cerebrospinal fluid. *Acinetobacter baumannii *was isolated mainly from bronchial lavage (100.0%) and graft samples (100.0%). *Pseudomonas aeruginosa* was found most notably in wound specimens (50.0%).

In 2023, *Klebsiella pneumoniae* was the most common isolate, particularly in urine, catheter, and sputum samples, each showing 100.0% detection. *Acinetobacter baumannii* was most commonly found in PTD extremity specimens (100.0%) and was also isolated from ascitic fluid. Pseudomonas aeruginosa appeared less frequently, primarily in blood and wound samples. Table 1 summarizes the information regarding the three most commonly encountered MDR isolates, *Klebsiella pneumoniae, Acinetobacter baumannii*, and *Pseudomonas aeruginosa*, across clinical specimen types from 2021 to 2023.

**Table 1:** The three most frequently isolated multidrug-resistant (MDR) pathogens, Acinetobacter baumannii, Klebsiella pneumoniae, and Pseudomonas aeruginosa, from various clinical specimen types collected between 2021 and 2023. Values represent the number of MDR isolates (n) followed by their percentage (%) of the total isolates of the same pathogen for that year and specimen type. PTD: percutaneous transluminal drainage

Year	Sample Type	Acinetobacter baumannii	Klebsiella pneumoniae	Pseudomonas aeruginosa
2021	Blood	1 (10.0%)	7 (70.0%)	2 (20.0%)
	Urine	–	3 (75.0%)	1 (25.0%)
	Pharyngeal Swab	1 (33.3%)	1 (33.3%)	1 (33.3%)
	Bronchial Lavage	2 (50.0%)	1 (25.0%)	1 (25.0%)
	Sputum	–	–	2 (100.0%)
	Catheter	2 (66.7%)	1 (33.3%)	–
	Wound	–	1 (100.0%)	–
	Pus	–	–	1 (100.0%)
	PTD Extremity	4 (57.1%)	3 (42.9%)	–
	CSF	–	–	–
	Peritoneal Fluid	–	–	–
	Ascitic Fluid	–	–	–
	Graft	–	–	–
2022	Blood	–	5 (83.3%)	1 (16.7%)
	Urine	–	4 (66.7%)	2 (33.3%)
	Pharyngeal Swab	1 (50.0%)	1 (50.0%)	–
	Bronchial Lavage	1 (100.0%)	–	–
	Sputum	–	–	–
	Catheter	1 (16.7%)	4 (66.7%)	1 (16.7%)
	Wound	1 (50.0%)	–	1 (50.0%)
	Pus	–	1 (100.0%)	–
	PTD Extremity	1 (33.3%)	2 (66.7%)	–
	CSF	–	1 (100.0%)	–
	Peritoneal Fluid	–	–	–
	Ascitic Fluid	–	–	–
	Graft	1 (100.0%)	–	–
	Other	–	1 (100.0%)	–
2023	Blood	1 (16.7%)	4 (66.7%)	1 (16.7%)
	Urine	–	6 (100.0%)	–
	Pharyngeal Swab	–	–	–
	Bronchial Lavage	–	–	–
	Sputum	–	1 (100.0%)	–
	Catheter	–	3 (100.0%)	–
	Wound	1 (50.0%)	1 (50.0%)	–
	Pus	–	–	–
	PTD Extremity	2 (100.0%)	–	–
	CSF	–	–	–
	Peritoneal Fluid	–	–	–
	Ascitic Fluid	1 (100.0%)	–	–
	Graft	–	–	–

Monthly distribution of MDR bacteria from 2021 to 2023

**Figure 3:**
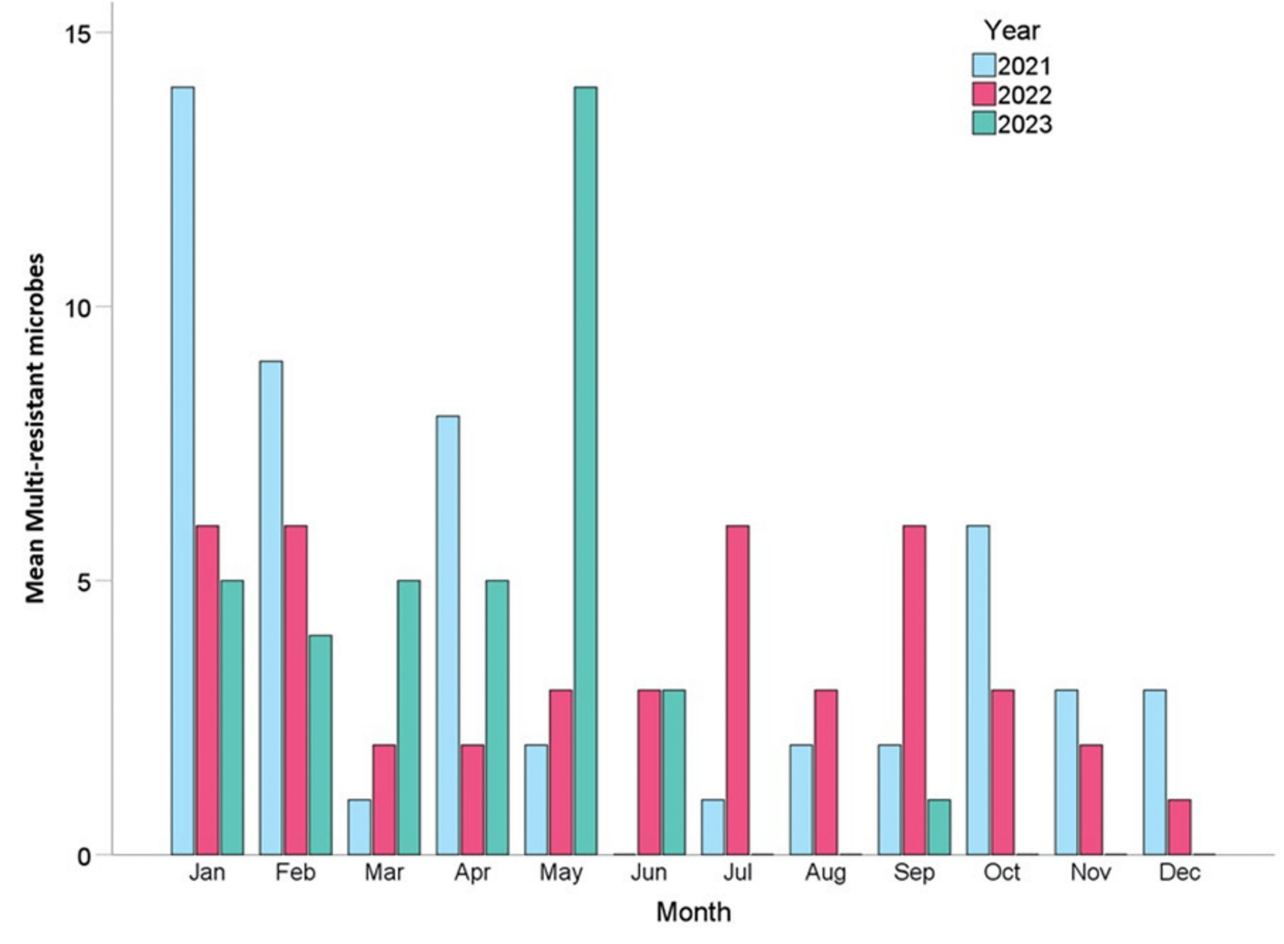
Monthly distribution of multidrug-resistant microbes by year

Figure 3 illustrates the monthly distribution of MDR microorganisms from 2021 to 2023. While the bar chart initially suggests noticeable fluctuations, such as a peak in January 2021 and another in May 2023, these apparent variations did not reach statistical significance. Analysis using the Kruskal-Wallis test revealed no significant differences in the monthly distribution of MDR isolates within each year. This result was consistent across all three years, with identical p-values of 0.443, indicating the absence of a seasonal pattern in MDR occurrence.

Department-wise distribution of MDR microorganisms

The distribution of multidrug-resistant organisms varied across hospital departments over the three-year period. *Klebsiella pneumoniae* is isolated more often in the Intensive Care Unit, Emergency Department, and general Surgery departments. Notably, it was also isolated in several specialized units, including the coronary care unit and departments of cardiology and oncology. *Acinetobacter baumannii* was isolated more in critical care settings, particularly in the COVID-designated ICU and standard ICU, as well as in departments managing complex cases such as neurosurgery and pulmonology. *Pseudomonas aeruginosa* appeared more sporadically but was repeatedly detected in surgical departments and the emergency department. While other organisms, such as *Proteus mirabilis*, *Stenotrophomonas maltophilia*, and *Providencia stuartii,* were isolated, their presence was recorded in both high-risk inpatient wards and outpatient settings. Overall, the data underscore the central role of ICUs and surgical departments as key reservoirs for MDR pathogens, reflecting both patient acuity and antimicrobial exposure. Table 2 shows the distribution of MDR isolates across various hospital departments. The ICU and surgery department exhibited the highest number of MDR isolates, accounting for 35% and 25% respectively, followed by the orthopedics department with 15%. This distribution highlights the concentrated presence of these pathogens in departments with high patient acuity and surgical activity.

**Table 2:** Distribution of multidrug-resistant (MDR) microorganisms by hospital department. The table displays the absolute number of isolates and the corresponding percentage of the total MDR isolates found in each department. Data are provided as numbers and total percentages (%).

Variable	2021						2022						2023			
Departments/Clinics	Acinetobacter baumannii	Klebsiella pneumoniae	Proteus mirabilis	Providencia stuartii	Pseudomonas aeruginosa	Stenotrophomonas maltophilia	Acinetobacter baumannii	Escherichia coli	Klebsiella pneumoniae	Proteus mirabilis	Providencia stuartii	Pseudomonas aeruginosa	Acinetobacter baumannii	Klebsiella pneumoniae	Providencia stuartii	Pseudomonas aeruginosa
Intensive Care Unit	5 (33.3)	9 (60.0)	-	-	-	1 (6.7)	3 (25.0)	-	9 (75.0)	-	-	-	2 (22.2)	7 (77.8)	-	-
Emergency Department	-	3 (75.0)	-	-	1 (25.0)	-	-	1 (12.5)	5 (62.5)	1 (12.5)	-	1 (12.5)	1 (7.1)	13 (92.9)	-	-
2^nd^ Department of Pathology	-	1 (20.0)	1 (20.0)	--	3 (60.0)	--	1 (25.0)		2 (50.0)	-	-	1 (25.0)	-	2 (66.7)	1 (33.3)	-
COVID-designated Intensive Care Unit	6 (75.0)	1 (12.5)	-	-	1 (12.5)	-	-	-	-	-	-	-	-	-	-	-
3^rd^ Department of Surgery	1 (20.0)	2 (40.0)	-	-	1 (20.0)	1 (20.0)	-	-	-	-	1 (100)	-	-	-	-	-
Department of Gastroenterology	-	-	-	-	-	-	-	-	2 (100)	-	-	-	-	2 (66.7)	-	1 (33.3)
Clinic of Infectious Diseases	1 (100)	-	-	-	-	-	-	1 (25.0)	3 (75.0)	-	-	-	-	1 (100)	-	-
Department of Neurosurgery	1 (33.3)	-	1 (33.3)	-	-	1 (33.3)	-	-	2 (100)	-	-	-	-	-	-	-
A Unit, Department of Pathology	-	-	-	-	1 (100)	-	-	-	2 (100)	-	-	-	-	3 (100)	-	-
Department of Pulmonology	1 (33.3)	1 (33.3)	-	1 (33.3)	v	-	-	-	-	-	-	1 (100)	-	-	-	-
Department of Hematology	-	-	-	-	1 (100)	-	-	-	-	-	-	-	-	1 (100)	-	-
Department of Plastic Surgery	-	1 (100)	-	-	-	-	-	-	-	-	-	1 (100)	-	-	-	-
A Unit, Department of Surgery	-	-	-	-	1 (100)	-	-	-	1 (100)	-	-	-	-	-	-	-
Department of Vascular Surgery	-	-	-	-	-	-	1 (100)	-	-	-	-	-	-	-	-	-
B Unit, Department of Surgery	-	-	-	-	-	-	-	-	-	-	-	-	-	1 (100)	-	-
Outpatient Clinic	-	1 (100)	-	-	-	-	-	-	-	-	-	-	-	-	-	-
Department of Cardiology	-	-	-	-	-	-	-	-	-	-	-	1 (100)	-	-	-	-
Department of Neurology	-	-	-	-	-	-	-	-	-	-	-	-	1 (100)	-	-	-
Department of Nephrology	-	-	-	-	-	-	-	-	1 (100)	-	-	-	-	-	-	-
Department of Oncology	-	1 (100)	-	-	-	-	-	-	-	-	-	-	-	-	-	-
Department of Rheumatology	-	-	-	-	-	-	-	-	-	-	-	-	-	1 (100)	-	-
Coronary Care Unit	-	-	-	-	-	-	-	-	1 (100)	-	-	-	-	-	-	-
Department of Oral & Maxillofacial Surgery	-	-	-	-	-	-	-	-	1 (100)	-	-	-	-	-	-	-
B Unit, Department of Orthopedic Surgery	-	-	-	-	-	-	-	-	-	-	-	-	-	1 (100)	-	-

Microorganism-wise trends in antimicrobial resistance for 2021, 2022, and 2023

Over the three-year period, *Klebsiella pneumoniae* emerged as the microorganism with the most extensive and evolving resistance profile. In 2021, resistance was already evident against several key antibiotics, particularly third-generation cephalosporins such as ceftriaxone and cefuroxime. By 2023, however, the organism had developed resistance to nearly all antibiotics tested.

*Acinetobacter baumannii* also showed significant resistance early on, especially in 2021, with sustained patterns in the following years.

*Pseudomonas aeruginosa* demonstrated a more stable resistance profile. While resistant isolates were present across all years, the extent of resistance was generally lower, and no major shifts in its pattern were observed.

**Figure 4:**
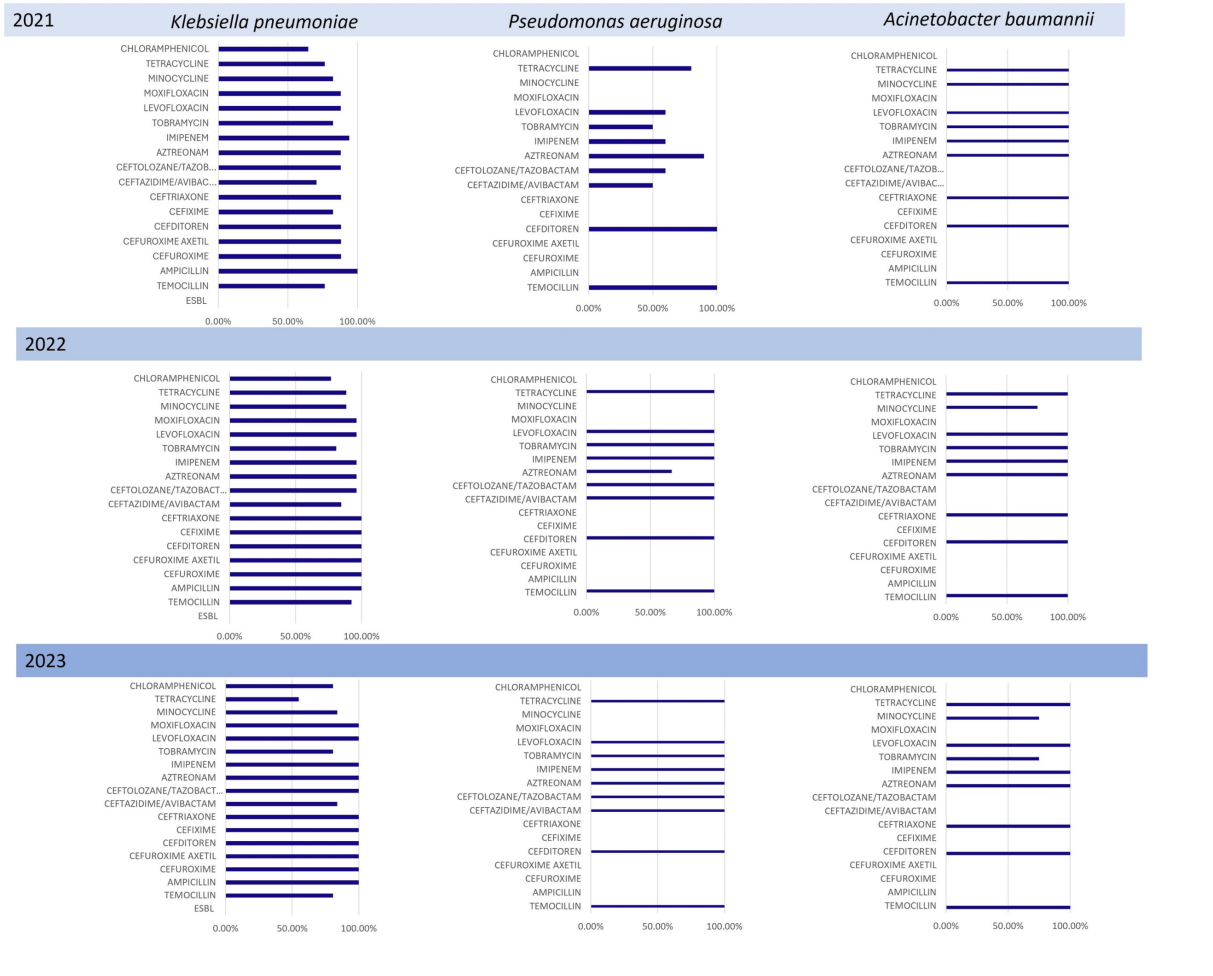
Trends in antimicrobial susceptibility by organism and year for the three most common organisms. The graph illustrates the percentage of antibiotic susceptibility for Klebsiella pneumoniae, Pseudomonas aeruginosa, and Acinetobacter baumannii on a year-by-year basis (2021, 2022, and 2023). Each bar represents an antibiotic class, and the numerical values indicate the percentage of isolates susceptible to that class. This allows for a clear visualization of changes in resistance over the study period.

Taken together, the findings point to *Klebsiella pneumoniae* as the organism that was most frequently identified and had the most notable increase in resistance over time, while *Acinetobacter baumannii* maintained its established resistance levels, and *Pseudomonas aeruginosa* remained relatively stable. Figure 4 illustrates the antibiotic susceptibility of these three most common microorganisms on a year-by-year basis.

## Discussion

The rise and spread of MDR microorganisms have become a major challenge for healthcare systems, limiting treatment options and increasing both morbidity and mortality. This problem is especially evident in hospital settings, where increased antimicrobial use and the presence of vulnerable patient groups create ideal conditions for resistance to develop [9, 19]. Previous studies have shown that MDR infections make treatment more difficult, prolong hospitalization, and raise overall healthcare costs [1, 10]. In Greece, antimicrobial resistance remains one of the most pressing public health concerns, with resistance rates among the highest in Europe [2, 19]. In this context, ongoing monitoring is critical to guide infection control and support more targeted antimicrobial use. This study adds to the existing literature by analyzing the occurrence and behavior of MDR pathogens over a three-year period in a tertiary military hospital, focusing on their distribution by department, type of clinical specimen, and resistance trends.

Based on the above results, it appears that the most frequently occurring MDR microorganism is *Klebsiella pneumoniae*, followed by *Acinetobacter baumannii*, a finding also reported by Jara et al. (2021) in their study on the prevalence of MDR microorganisms in two hospitals in Brazil [10]. Our findings, particularly the high prevalence of *Klebsiella pneumoniae* and *Acinetobacter baumannii *as MDR pathogens, are consistent with data from other regional hospitals in Northern Greece, such as the one described by Tsalidou et al. (2023) [23]. MDR microorganisms were detected most often in clinical urine samples, indicating that the urinary tract is the most common site for the development of such infections within our study cohort. This finding is consistent with the established knowledge that urinary tract infections are a prevalent type of infection in hospital settings, but our analysis specifically highlights the high prevalence of MDR organisms within these infections. This observation aligns with the research by Martins et al. (2004), who studied ICU patients in a hospital in São Paulo [24].

The department with the highest number of MDR microorganisms was the ICU, a finding similarly reported by Andrade, Leopoldo, and Haas (2006), in their study conducted in a Brazilian hospital [1]. However, this differs from the more recent study by Ioannou et al. (2022) in eight public hospitals in Crete, where the pathology clinic was the most frequently affected department [9]. The findings from our study, where the pathology clinic was not the most frequently affected department, are more aligned with the results reported from a regional hospital in Northern Greece by Tsalidou et al. (2023), where the ICU was the most common site for MDR isolates [21].

*Klebsiella pneumoniae* was found at a higher rate in 2023 compared to the other two years of the study, whereas Polemis et al. (2021) reported a higher incidence of *Acinetobacter baumannii *than what was recorded by the Hellenic Electronic Microbial Resistance Surveillance System [19]. Throughout the three-year period, *Klebsiella pneumoniae* consistently appeared as the leading MDR organism in blood and urine specimens. *Acinetobacter baumannii *was more often associated with respiratory and soft tissue infections, while *Pseudomonas aeruginosa *showed a more limited and sporadic presence, typically confined to specific specimen types, including sputum and trauma-related wound swabs.

One notable finding in this study is the absence of MDR isolates during the second half of 2023. The absence of MDR isolates during the second half of 2023 is a notable finding. This change is not attributed to a shift in the bacterial susceptibility patterns but rather to internal changes in the hospital's diagnostic and reporting practices, which may have impacted the detection or documentation of MDR cases during that specific period. This underscores the importance of consistent methodologies in surveillance studies.

While the high resistance profile of *Acinetobacter baumannii *remained stable over the study period, its continued presence is a significant clinical concern, especially in critical care settings. In contrast, *Pseudomonas aeruginosa* did not show the notable increase in resistance that was observed for *Klebsiella pneumoniae*.

Despite limited knowledge about the optimal therapeutic management of these infections, the results suggest that some antibiotic combinations are currently effective against certain MDR microorganisms. However, a standardized treatment approach has not yet been established. The incidence of MDR microorganisms was notable throughout the three years studied, and their presence was confirmed in a wide range of clinical specimens. Our findings indicate that the majority of MDR organisms were identified in samples from ICU patients, underscoring the need for targeted interventions in high-acuity clinical settings. In light of these findings, addressing this problem requires a multifaceted approach. Improved infection control protocols, particularly in the ICU, are necessary. Furthermore, the most crucial step remains the reduction of inappropriate antimicrobial use, which can be achieved through effective antimicrobial stewardship programs that promote the appropriate selection, dosing, and duration of antibiotic therapy.

It is also important to expand on these findings by investigating the duration of hospitalization before the appearance of MDR infections in order to determine whether they were acquired during the hospital stay. Furthermore, it would be useful to examine the possibility of patient-to-patient transmission, especially in cases where MDR isolates originated from the same department during the same time period.

Limitations

This study has certain limitations that should be acknowledged. First, the study was conducted in a single tertiary care military hospital. Although the patients, consisting of armed forces, law enforcement, fire services, and their families, live within the general population, the unique clinical environment and patient demographics of a specialized institution may still limit the direct generalizability of the findings to broader community settings. Nonetheless, these findings provide valuable insights into antimicrobial resistance trends in similar specialized healthcare environments. No molecular testing was performed to identify specific resistance genes, so we cannot comment on the genetic mechanisms behind the observed resistance patterns. Also, because the data were based on clinical samples, it is possible that some cases, especially colonizations or infections that were not cultured, were missed. Furthermore, due to the anonymized nature of the dataset, we were unable to analyze the co-occurrence of multiple MDR pathogens per patient or to evaluate patient-related factors such as age, sex, underlying conditions, and prior antibiotic exposure, all of which are critical for identifying risk profiles. This limitation also prevented us from investigating the timing of MDR infection acquisition or the possibility of patient-to-patient transmission. Despite these limitations, the study provides valuable insights into local antimicrobial resistance trends and highlights the critical need for ongoing surveillance and targeted interventions to address this growing public health threat.

## Conclusions

Based on the findings of this study, infections caused by MDR microorganisms remain a serious public health concern. They pose a constant challenge for both patients and healthcare professionals, as high rates of MDR pathogens were identified in clinical samples over the three-year period. *Klebsiella pneumoniae* was the most common MDR organism, followed by *Acinetobacter baumannii* and Pseudomonas aeruginosa. Other organisms, such as* Stenotrophomonas maltophilia*, *Proteus mirabilis*, *Providencia stuartii*, and *Escherichia coli*, were detected less frequently. Urinary tract infections were the most common type of infection, followed by bloodstream infections. The majority of MDR isolates originated from the ICU and trauma ICU, highlighting the increased risk in these departments. In some cases, combinations of antibiotics showed effectiveness against MDR strains, but no standard treatment protocol has yet been established. Therefore, the careful and rational use of antibiotics, along with improved antiseptic techniques and more consistent infection control measures, are essential first step in addressing this growing issue.

## Appendices

Appendix A 

**Table 3:** Monthly microbial detection data with relative percentages. Count (percentage relative to total samples) (percentage relative to multi-resistant isolates). When there are no multi-resistant isolates in a month, the cell is blank.

Year	Month	Total samples	Multi-resistant	*Pseudomonas aeruginosa*	*Klebsiella pneumoniae*	Acinetobacter baumannii	*Stenotrophomonas maltophilia*	Proteus mirabilis	*Providencia stuartii*	*Escherichia coli*
2021	Jan	208	14	3 (1.44%) (21.43%)	5 (2.40%) (35.71%)	4 (1.92%) (28.57%)	1 (0.48%) (7.14%)	1 (0.48%) (7.14%)		
	Feb	158	9	3 (1.90%) (33.33%)	3 (1.90%) (33.33%)	3 (1.90%) (33.33%)				
	Mar	166	1			1 (0.60%) (100.00%)				
	Apr	233	8	2 (0.86%) (25.00%)	3 (1.29%) (37.50%)	3 (1.29%) (37.50%)				
	May	186	2			2 (1.08%) (100.00%)				
	Jun	156	0							
	Jul	178	1		1 (0.56%) (100.00%)					
	Aug	208	2	1 (0.48%) (50.00%)	1 (0.48%) (50.00%)					
	Sep	246	2	1 (0.41%) (50.00%)				1 (0.41%) (50.00%)		
	Oct	183	6		3 (1.64%) (50.00%)	1 (0.55%) (16.67%)	1 (0.55%) (16.67%)	1 (0.55%) (16.67%)		
	Nov	218	3		3 (1.38%) (100.00%)					
	Dec	175	3		1 (0.57%) (33.33%)	1 (0.57%) (33.33%)				1 (0.57%) (33.33%)
2022	Jan	181	6		4 (2.21%) (66.67%)	2 (1.10%) (33.33%)				
	Feb	185	6	1 (0.54%) (16.67%)	4 (2.16%) (66.67%)				1 (0.54%) (16.67%)	
	Mar	202	2		2 (0.99%) (100.00%)					
	Apr	188	2	1 (0.53%) (50.00%)	1 (0.53%) (50.00%)					
	May	208	3	1 (0.48%) (33.33%)	1 (0.48%) (33.33%)					1 (0.48%) (33.33%)
	Jun	182	3		3 (1.65%) (100.00%)					
	Jul	180	6	2 (1.11%) (33.33%)	3 (1.67%) (50.00%)	1 (0.56%) (16.67%)				
	Aug	215	3		2 (0.93%) (66.67%)			1 (0.47%) (33.33%)		
	Sep	199	6		4 (2.01%) (66.67%)	1 (0.50%) (16.67%)				1 (0.50%) (16.67%)
	Oct	190	3		3 (1.58%) (100.00%)					
	Nov	182	2		1 (0.55%) (50.00%)	1 (0.55%) (50.00%)				
	Dec	215	1		1 (0.47%) (100.00%)					
2023	Jan	254	5		4 (1.57%) (80.00%)	1 (0.39%) (20.00%)				
	Feb	217	4		4 (1.84%) (100.00%)					
	Mar	225	5		3 (1.33%) (60.00%)	1 (0.44%) (20.00%)				1 (0.44%) (20.00%)
	Apr	212	5		4 (1.89%) (80.00%)	1 (0.47%) (20.00%)				
	May	303	14	1 (0.33%) (7.14%)	12 (3.96%) (85.71%)	1 (0.33%) (7.14%)				
	Jun	249	3		3 (1.20%) (100.00%)					
	Jul	236	0							
	Aug	336	0							
	Sep	338	1		1 (0.30%) (100.00%)					
	Oct	322	0							
	Nov	270	0							
	Dec	252	0							
